# Range of ventricular dimensions and function by steady-state free precession cine cardiac magnetic resonance in patients late after the Fontan operation

**DOI:** 10.1186/1532-429X-14-S1-P105

**Published:** 2012-02-01

**Authors:** Rahul H Rathod, Yuli Y Kim, Ashwin Prakash, Ioannis Germanakis, Andrew J Powell, Tal Geva

**Affiliations:** 1 Department of Cardiology, Children's Hospital Boston, Boston, MA, USA; 2 Department of Cardiology, Hospital of the University of Pennsylvania and Children’s Hospital of Philadelphia, Philadelphia, PA, USA; 3 Department of Pediatrics, University Hospital Heraklion, Heraklion, Greece

## Summary

To characterize the range of ventricular size and function evaluated by steady-state free precession (SSFP) cine cardiac magnetic resonance (CMR) in functional single ventricle (FSV) patients late after Fontan operation and to identify factors associated with FSV dysfunction.

## Background

Ventricular size and function is important in patients with FSV after Fontan and adverse remodeling has been associated with poor clinical outcomes. Due to the limitations of echocardiography and other noninvasive techniques, CMR remains the gold standard for the assessment of ventricular size and function. However, the range and distribution of FSV size and function in this population has not been characterized in a large cohort across a broad range of ages using contemporary SSFP techniques.

## Methods

All patients at our center following a Fontan operation who had a CMR study from 4/2002 to 1/2011 were retrospectively reviewed. In patients with multiple CMR studies, the most recent study was used for analysis. If 2 ventricles were present and connected to the systemic circulation, the end-diastolic (EDV) and end-systolic (ESV) volumes were summated to describe total effective volumetric and function data. Examinations were performed on a 1.5 T scanner using a standardized, previously published imaging protocol. Combined EDV_i_, ESV_i_, and ventricular mass index (Mass_i_) were indexed to BSA^1.3^. Other clinically relevant data including demographic information, cardiac diagnoses, surgical history, and clinical symptoms and outcomes were recorded. Ventricular dysfunction was defined as ejection fraction (EF) ≤ 45% and variables associated with FSV dysfunction were analyzed.

## Results

Of the 261 patients who had a CMR study after the Fontan operation, 46 (18%) had incomplete data due to metallic artifacts from coils and other devices. The median age at CMR of the remaining 215 patients was 18.3 years [13.6, 26.1], age at Fontan 3.6 years [2.3, 7.1], and the median time since Fontan was 14.6 years [9.5, 19.3]. LV morphology was present in 45% of patients, RV morphology in 30%, and biventricular in 25%. The lateral tunnel Fontan operation was performed in 70% of patients, right atrium to pulmonary artery in 21%, extracardiac in 5%, and right atrium to right ventricle in 4%. The medians, interquartile range (IQR), means, standard deviations (SD), and ranges of FSV size and function are summarized in Table 1. Compared with patients without FSV dysfunction (n=174), those with dysfunction (n=41) were older at the time of Fontan (4.9 v. 3.4 years, *p*=0.026), older at time of CMR (22.3 v. 17.4 years, *p*=0.02), and had higher frequencies of ≥moderate atrioventricular valve regurgitation (27% v. 7.5%, *p*=0.001), non-sustained ventricular tachycardia (27% v. 12%, *p*=0.023), and stroke (32% v. 16%, *p*=0.025).

**Table 1:** SSFP measurements of ventricular size and function in Fontan patients

CMR Measurement	Median [IQR]	Mean ± SD	Range
EDV_i_ (ml/BSA^1.3^)	94 [76, 116]	94 ± 44	39-359
ESV_i_ (ml/BSA^1.3^)	42 [32, 57]	50 ± 34	11-277
EF (%)	55 [47, 61]	54 ± 10	13-78
Mass_i_ (g/BSA^1.3^)	55 [43, 68]	60 ± 24	17-181
Mass:Volume ratio	0.57 [0.48, 0.69]	0.63 ± 0.26	0.25-2.3

## Conclusions

This study provides the range and distribution of ventricular size and function in a large cohort of patients late after the Fontan operation using contemporary CMR techniques and demonstrates important variations in those patients with significant systolic dysfunction.

## Funding

None.

